# Social determinants of leprosy in a hyperendemic State in North Brazil

**DOI:** 10.1590/S1518-8787.2017051006655

**Published:** 2017-07-07

**Authors:** Lorena Dias Monteiro, Rosa Maria Salani Mota, Francisco Rogerlândio Martins-Melo, Carlos Henrique Alencar, Jorg Heukelbach

**Affiliations:** I Departamento de Saúde Comunitária. Faculdade de Medicina. Universidade Federal do Ceará. Fortaleza, CE, Brasil; II Departamento de Estatística e Matemática Aplicada. Universidade Federal do Ceará. Fortaleza, CE, Brasil; III Secretaria de Estado da Saúde do Tocantins. Palmas, TO, Brasil; IV Instituto Federal de Educação, Ciência e Tecnologia do Ceará. Caucaia, CE, Brasil; V School of Public Health. Tropical Medicine and Rehabilitation Sciences. James Cook University. Townsville, Australia

**Keywords:** Leprosy, epidemiology, Socioeconomic Factors, Neglected Diseases, prevention & control, Health Inequalities

## Abstract

**OBJECTIVE:**

To identify the socioeconomic, demographic, operational, and health service-related factors associated with the occurrence of leprosy in a hyperendemic State in North Brazil.

**METHODS:**

This is an ecological study based on secondary data from the *Sistema de Informações de Agravos de Notificação* in municipalities of the State of Tocantins from 2001 to 2012. Units of analysis were the 139 municipalities of the State. Negative binomial log linear regression models were used to estimate incidence rate ratios.

**RESULTS:**

In bivariate analysis, the incidence rate ratios were significantly higher for municipalities with higher income ratio of the poorest 20.0% (1.47; 95%CI 1.19–1.81) and better Municipal Human Development Index (1.53; 95%CI 1.14–2.06). In multivariate analysis, the incidence rate ratios were significantly higher in municipalities with higher proportion of immigrants (1.31; 95%CI 1.11–1.55) and higher proportion of households with waste collection (1.37; 95%CI 1.11–1.69). There was a significant reduction in the incidence rate ratio with increased coverage of the *Bolsa Família* Program (0.98; 95%CI 0.96–0.99).

**CONCLUSIONS:**

Control programs need to focus on activities in municipalities of greater social vulnerability with intersectoral investment for the improvement of the living conditions of the population.

## RESUMO

**OBJETIVO:**

Identificar fatores socioeconômicos, demográficos, operacionais e de serviços de saúde associados à ocorrência da hanseníase em um estado hiperendêmico do norte do Brasil.

**MÉTODOS:**

Estudo ecológico com dados secundários do Sistema de Informações de Agravos de Notificação em municípios do estado do Tocantins de 2001 a 2012. As unidades de análise foram os 139 municípios do estado. Modelos de regressão log linear binomial negativa foram utilizados para estimar as razões de taxas de incidência.

**RESULTADOS:**

Na análise bivariada, a razão de taxa de incidência foi significativamente maior para os municípios com maior razão de renda dos 20,0% mais pobres (1,47; IC95% 1,19–1,81) e melhor Índice de Desenvolvimento Humano Municipal (1,53; IC95% 1,14–2,06). Na múltipla, a razão de taxa de incidência foi significativamente superior em municípios com maior concentração de imigrantes (1,31; IC95% 1,11–1,55) e proporção de domicílios com coleta de lixo (1,37; IC95% 1,11–1,69). Houve redução significativa da razão de taxa de incidência com o aumento da cobertura do programa bolsa família (0,98; IC95% 0,96–0,99).

**CONCLUSÕES:**

Os programas de controle precisam focar as atividades em municípios de maior vulnerabilidade social com investimentos intersetoriais para a melhoria das condições de vida da população.

## INTRODUCTION

Despite significant improvements in the control of leprosy in recent decades, the disease persists as a public health problem in many countries in the world, including Brazil^29^. Leprosy is a Neglected Tropical Disease (NTD) with unequal occurrence, mainly in socioeconomically disadvantaged and marginalized populations in tropical countries^11^.

Approximately 233,000 new leprosy cases were recorded in the world in 2013. The Americas recorded about 17% all cases, with 92% of these from Brazil. The overall case detection rate in the country was 15.4 new cases per 100,000 inhabitants^29^. The distribution of the disease is heterogeneous in Brazil, with new cases concentrated in the poorest regions of the country (North, Midwest and Northeast)^1,24^. Despite advances in the control of leprosy at the national level in recent years, the detection rates are still high in these regions^1^.

The State of Tocantins, located in the North region of the country, ranked second in the Brazilian States in terms of new cases per 100,000 inhabitants (60.9) and ranked first in those aged 15 years or less (19.7) in 2013^16,a^. This epidemiological situation indicates a relationship of the endemic process of the disease with the habitation of new regions, as Tocantins is a State with a large area of agricultural borders. Migratory movements and population growth leading to deforestation seem to promote an increased incidence of the disease^18^. Additionally, the high values of the indicators may reflect the social vulnerability of the disease, which also favors transmission and endemicity of the bacillus^7,14^.

The understanding of the different determinants and conditions of the disease in that territory can support local programs to control the disease. The objective of this study was to identify socioeconomic, demographic, operational, and health service-related factors associated with the occurrence of leprosy in a hyperendemic State in North Brazil.

## METHODS

This study is part of a major project of the Universidade Federal do Ceará called INTEGRAHANS – Norte/Nordeste. The project is an integrated approach on epidemiological, clinical, psychosocial, and operational patterns of leprosy in the States of Tocantins, Rondônia, and Bahia.

Tocantins is located in the North region of Brazil (Figure). It is the most recent State in the country (founded in 1988) and part of the Brazilian Amazon region, with a predominantly savannah (*Cerrado*) vegetation. It has a territorial extension of 277,622 km^2^ and an estimated population of 1.5 million in 2014. It is divided into eight health regions. The urbanization rate of the State increased from 40.0% in 1980 to 79.0% in 2010.

**Figure:**
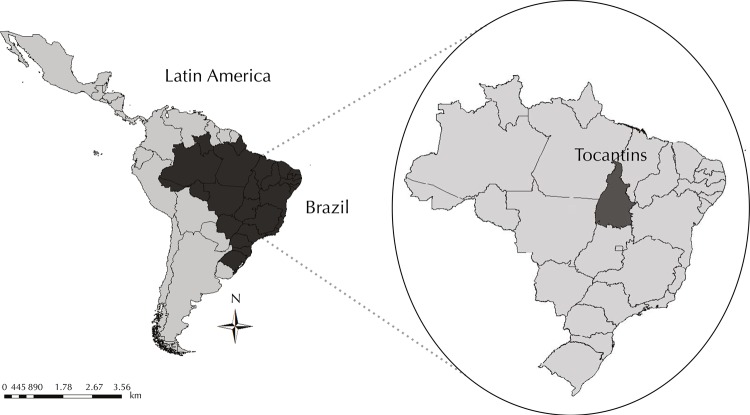
Map of the Latin America, Brazil, and State of Tocantins.

Sociodemographic data point to a significant improvement of the living conditions of the population in the last two decades. The Municipal Human Development Index (MHDI) increased from 0.369, in 1991, to 0.699, in 2010. The proportion of extreme poverty was reduced from 31.8%, in 1991, to 10.2%, in 2010. Income inequality pointed by the Gini index ranged from 0.63, in 1991, to 0.60, in 2010. Average *per capita* income increased by 140.8% in the last two decades, from R$243.58, in 1991, to R$586.62, in 2010 (income adjusted for inflation). The proportion of poverty, i.e., individuals with income *per capita* of less than R$140.00 (August 2010 figures), deceased from 59.1%, in 1991, to 22.2%, in 2010. The illiteracy rate of persons aged 15 years or more decreased from 31.4%, in 1991, to 13.1%, in 2010. There was a significant growth of households with running water and sanitation, increasing from 29.0%, in 1991, to 94.3%, in 2010. The proportion of the population with waste collection in the urban area of the municipalities, increased from 77.3%, in 1991, to 97.0%, in 2010^b^.

We performed an ecological study including data from the 139 municipalities of the State as units of analysis (2001–2012). There were 14,532 new leprosy cases during the study period. We did not consider records with diagnostic error, duplicate entries, unknown municipality, and individuals living in other States before the calculation of indicators.

The data of leprosy cases were obtained from the *Sistema Nacional de Agravos de Notificação* (SINAN – National System of Reportable Diseases) of the Ministry of Health, based on compulsory notification records. These records are standardized with sociodemographic and clinical information provided by health professionals^c^. The operational indicators used included the average percentage of contacts of leprosy cases among those registered from 2001 to 2012, and the average proportion of cases with grade 2 disability among new cases from 2001 to 2012.

Population, demographic, and socioeconomic data were obtained from the Brazilian Institute of Geography and Statistics (IBGE). Population data were based on data from the national census (2010), with estimates for the intercensus years (2001–2009 and 2011–2012)^b^.

Data regarding the program of income transfer (*Bolsa Família* Program) in 2010 were obtained from the Ministry of Social Development – MDS (*Programa Bolsa Famíla* – BFP)^d^. Data related to municipal health services in 2010 were acquired from the *Departamento da Atenção Básica* (DAB – Department of Primary Health Care) of the Ministry of Health^e^. These data are available in the public domain.

We calculated the smoothed detection rates (per 100,000 inhabitants) as outcome using the empirical Bayesian method. Smoothing was adopted to reduce the fluctuations of crude rates associated with the small areas, using the information of the neighboring municipalities^3^.

The independent variables included the demographic and socioeconomic characteristics of the municipalities, as well as the variables related to the government program of income transfer and health services. In reference to a national study^7^, the following characteristics of the municipalities were obtained from the 2010 Census: Municipal Human Development Index (MHDI), illiteracy rate, proportion of low-income population (half minimum wage/nominal household monthly income *per capita* < R$255.00), proportion of population living in extreme poverty (nominal household monthly income *per capita* < R$70.00), average household monthly income *per capita*, urbanization rate, gender ratio, unemployment rate, Gini index, income ratio (income ratio of the richest 20.0% and the poorest 20.0%), income ratio of the poorest 20.0% (income ratio of the 20.0% with better and 20.0% with worse income among the poor), proportion of households with two or more persons per bedroom, proportion of households with inadequate sanitation (no connection to the network of water supply, sanitation, or had no access to waste collection), proportion of immigrants with permanent residency in the municipality during the past 10 years, proportion of households with running water and bathroom, proportion of households with waste collection^b^. The variable for the income transfer program was: proportion of coverage of the *Bolsa Família* Program (BFP) in 2010. According to the methodology of Rasella et al. (2013), this variable was calculated dividing the number of individuals in the BFP (multiplication of the number of families benefited by the average size of the family) by the total population of the same municipality^25^.

The variables related to health services of the municipalities included: average monthly percentage of population covered by the Family Health Strategy (FHS) in 2010^e^ and operational indicators of the SINAN – average proportion of contacts of cases of leprosy examined among the records of 2001 and 2012 and average proportion of cases with grade 2 disability among new cases assessed from 2001 to 2012.

Continuous variables were categorized into approximate quartiles. However, for the variable “proportion of cases with grade 2 disability among new cases assessed”, we used the categories for the parameters of interpretation of this indicator: low (< 5.0%), average (5.0% to < 10.0%), and high (≥ 10.0%), as recommended by the Ministry of Health. The distributions of the values of the smoothed detection rates of leprosy, according to the characteristics of the municipalities, were described based on the values of the medians, minimum, and maximum. We analyzed the association between the smoothed detection rates with socioeconomic, demographic, income transfer, and health service conditions in the municipalities of the State of Tocantins.

The distribution of the detection rates in the period showed asymmetry to the right (positive asymmetric distribution of 1.089 and with extreme values, Kurtosis = 2.242). Therefore, we decided to use the hierarchical log-linear model with negative binomial response in the analytical step to identify which parameters significantly determine the variation of the number of cases of the disease by municipality. The test of significance of the parameters of the rates was carried out by Wald statistic, and we estimated the Incidence Rate Ratio (IRR) of the risk factors for the disease with their respective 95% confidence intervals (95%CI).

We estimated the Pearson correlation coefficient to avoid multicolinearity in the multivariate regression analysis. For the multivariate model, we selected the variables that, in the bivariate regression, were related with the outcome at a level of significance of up to 10.0%, and in accordance with the best quantitative or qualitative fit.

We adapted the hierarchical theoretical model proposed by Victora et al.^28^ to identify the factors associated with the detection rates of new cases of leprosy. The independent variables were separated into proximal (income transfer program and health services) and distal levels (demographic and socioeconomic factors). We started hierarchical modeling by introducing all first-level (distal) variables at once. The significant variables in the analysis of the first level (p < 0.05) remained in the model and were part of the adjustment of the next level (proximal)^28^.

We used the backward elimination method and Wald statistic in each of the hierarchical levels for the construction of the final models. The corrected Akaike criterion was used to select the best model. The residues for the log-linear negative binomial model were examined using the probability distribution^22^ to assess the quality of the fit of the model.

The analyses were carried out using the Stata statistical program package version 11.2 (StataCorp, College Station, TX, USA) and ArcGis version 9.2 (Esri, Redlands, CA, USA) (ESRI, 2010).

The study was approved by the Ethical Review Board of the Universidade Federal do Ceará (No. 544,962 of Feb 28, 2014).

## RESULTS

The average annual smoothed detection rate in the period was 84.6 cases/100,000 inhabitants. The values ranged between 21.9 and 250.5 cases/100,000 inhabitants by municipality.

In the bivariate analysis, IRR was significantly higher for the municipalities with the lowest (< 25.1%) proportion of the population covered by the BFP compared with the highest proportion of coverage (25.1% to 33.9% and greater than 33.9%). Of the variables on income, only the income ratio of the poorest 20.0% presented significant IRR for municipalities with inequality above 3.4%. The IRR of the disease was higher for municipalities with detection of early cases, i.e., < 5.0% of cases with grade 2 disability. The municipalities with the highest concentration of immigrants with permanent residency in the last 10 years had the highest IRR (1.32; 95%CI 1.10–1.59) (Table 1).

**Table 1:** Bivariate analysis of selected variables by municipalities in the State of Tocantins, Brazil, 2001–2012.

Variable	Smoothed detection rate of leprosy per 100,000 inhabitants					

	Median	Minimum	Maximum	IRR	95%CI	*p*
Proportion of households with two or more persons per bedroom						
< 25.6	92.8	21.9	250.5	1	-	-
25.6 to 39.9	82.6	25.0	177.1	0.850	0.712–1.014	0.071
> 39.9	61.8	22.7	125.5	0.668	0.544–0.821	< 0.001
Income *per capita* of up to R$70.00 per household						
< 24.7	95.8	42.1	175.4	1.216	0.962–1.536	0.102
24.7 to 32.9	71.8	21.9	250.5	0.933	0.771–1.129	0.477
> 32.9	76.5	22.7	158.7	1	-	-
Income *per capita* of up to half minimum wage (R$255.00) per household						
< 130.9	71.8	21.9	138.8	0.672	0.565–0.799	< 0.001
130.9 to 146.6	88.0	25.0	155.4	0.841	0.689–1.027	0.089
> 146.6	88.4	24.2	250.5	1	-	-
Proportion of illiterates aged 15 or more per household						
< 15.2	85.3	31.9	250.5	1	-	-
15.2 to 20.7	81.3	21.9	181.4	0.827	0.693–0.987	0.036
> 20.7	56.1	22.7	125.5	0.600	0.488–0.737	< 0.001
Proportion of unemployed persons aged 16 or more per household						
< 5.4	84.9	22.7	223.3	1	-	-
5.4 to 9.6	83.1	21.9	250.5	1.009	0.829–1.229	0.926
> 9.6	68.5	25.0	155.4	0.844	0.669–1.065	0.153
Income ratio (20%–20%)						
< 18.5	91.8	24.2	223.3	1	-	-
18.5 to 25.9	80.4	25.0	250.5	0.788	0.655–0.949	0.012
> 25.9	74.7	22.7	177.1	0.715	0.577–0.885	0.002
Income ration of the poorest 20%						
< 1.7	74.2	21.9	120.7	1	-	-
1.7 to 3.4	78.0	22.7	250.5	1.129	0.940–1.356	0.194
> 3.4	95.2	24.2	223.3	1.472	1.194–1.813	< 0.001
Average income *per capita* per household						
< 293.5	66.7	22.7	125.5	0.689	0.561–0.846	< 0.001
293.5 to 423.4	80.9	21.9	181.4	0.781	0.654–0.934	0.007
> 423.4	93.2	31.9	250.5	1	-	-
Gini index						
< 0.5	83.4	24.2	223.3	1.262	0.976–1.631	0.076
0.5 to 0.6	81.4	22.7	177.1	1.075	0.879-1.315	0.480
> 0.6	74.8	21.9	250.5	1	-	-
Municipal Human Development Index						
< 0.6	66.7	22.7	125.5	1	-	-
0.6 to 0.7	82.2	21.9	181.4	1.168	1.142–2.061	0.114
> 0.7	81.7	31.9	250.5	1.534	1.142–2.061	0.004
Men to women ratio in the household						
< 104.6	74.8	24.2	250.5	1	-	-
104.6 to 111.8	79.1	21.9	181.4	0.932	0.768–1.13	0.471
> 111.8	85.3	22.7	155.1	0.962	0.773–1.196	0.725
Proportion of households in the urban area						
< 54.2	84.9	30.1	141.7	1	-	-
54.2 to 80.1	79.1	21.9	181.4	0.977	0.810–1.178	0.805
> 80.1	81.8	24.2	250.5	1.149	0.924–1.428	0.212
Proportion of immigrants with fixed residence per municipality in the last 10 years						
< 17.8	74.7	21.9	175.4	1		
17.8 to 23.9	92.6	30.1	250.5	1.306	1.089–1.565	0.003
> 23.9	96.7	31.2	181.4	1.323	1.103–1.586	0.004
Proportion of households with inadequate water supply and sanitation						
< 5.2	78.2	21.9	250.5	1	-	-
5.2 to 14.1	87.0	29.0	177.1	1.015	0.837–1.230	0.883
> 14.1	82.2	22.7	175.4	0.902	0.723–1.126	0.364
Proportion of household with running water and bathroom						
< 61.7	64.1	22.7	138.8	0.627	0.512–0.769	< 0.001
61.7 to 84.2	78.3	21.9	181.4	0.734	0.616–0.874	0.001
> 84.2	94.5	31.9	250.5	1	-	-
Proportion of households with waste collection						
> 97.7	98.6	97.8	99.8	1.583	1.284–1.952	0.000
88.8 to 97.7	95.3	89.3	97.7	1.432	1.196–1.714	0.000
> 88.8	81.2	32.3	88.8	1	-	-
Proportion of cases with grade 2 disability among those assessed						
< 5.0	85.7	21.9	250.5	1.383	1.067–1.794	0.014
5.0 to 10.0	81.3	29.1	158.7	1.329	1.002–1.761	0.048
> 10.0	62.9	30.2	120.5	1	-	-
Percentage of contacts examined among those assessed						
< 57.6	78.2	22.7	181.4	1	-	-
57.6 to 82.5	80.8	21.9	223.3	1.013	0.838–1.224	0.894
> 82.5	93.2	39.4	250.5	0.869	0.697–1.082	0.209
Average percentage of population covered by the Family Health Strategy per municipality						
< 73.3	74.6	25.0	175.4	1	-	-
73.3 to 90.6	81.2	21.9	250.5	1.175	0.968–1.425	0.103
> 90.6	85.1	31.9	181.4	1.213	0.973–1.513	0.086
Percentage of population covered by the *Bolsa Família* Program						
< 25.1	104.7	24.2	250.5	1.658	1.036–2.022	< 0.001
25.1 to 33.9	78.0	21.9	181.4	1.182	0.995–1.405	0.057
> 33.9	59.8	22.7	125.5	1	-	-

Socioeconomic and demographic variables, such as the proportion of households with two or more persons per bedroom, *per capita* income of up to half wage per household, and others, were significantly lower for leprosy (p < 0.05). The detection rates of these variables showed the highest median in the most vulnerable groups (Table 1).

In multivariate analysis, IRR was significantly higher in the municipalities with higher proportion of households with waste collection and higher proportion of immigrants (Table 2).

**Table 2:** Multivariate analysis of the average smoothed detection rate of leprosy (per 100,000 inhabitants) and selected variables, according to municipalities. State of Tocantins, Brazil, 2001–2012.

Variable	Coefficient	Standard error	IRR	95%CI	p
Distal variables – Level 1 adjustment					

Proportion of households with waste collection					
< 88.8	-	-	1	-	-
88.8 to 97.7	0.207	0.095	1.230	1.021–1.482	0.030
> 97.7	0.314	0.107	1.369	1.11–1.69	0.003
Proportion of immigrants with residence in the State during the last 10 years					
< 17.8	-	-	1	-	-
17.8 to 23.9	0.149	0.088	1.161	0.977–1.38	0.090
> 23.9	0.273	0.084	1.314	1.114–1.551	0.001
Unemployment rate per household in the population aged 16 years or more	-0.021	0.010	0.980	0.961–0.998	0.033
Proportion of municipalities with available potable water and bathroom	0.008	0.003	1.008	1.000–1.013	0.005

Proximal variables – Level 2 adjustment					

Proportion of persons registered in the *Bolsa Família* Program per household	-0.021	0.007	0.980	0.967–0.993	0.002

Increased coverage of the BFP was significantly associated with a reduced leprosy detection rate. This effect was maintained after controlling for sociodemographic and income variables. The municipalities with the highest unemployment rates per household in the population aged 16 years or more presented lower IRR (Table 2).

## DISCUSSION

This study indicates a significantly positive impact of public social policies in reducing the detection of leprosy incidence in municipalities in the State of Tocantins. The IRR was significantly higher in municipalities with higher ratio of income of the poorest 20.0%, better Municipal Human Development Index (MHDI), higher proportion of immigrants, and higher proportion of households with waste collection. There was a significant reduction of the IRR with increased coverage of BFP.

Social inequities determine the persistence and the difficulties of controlling NTD, causing higher vulnerability and risk for these diseases^11^. Poverty is one of the determining factors for the occurrence and transmission of leprosy^7,13,14^. In this study, the variables related to poverty were clearly associated with the high incidence.

On the other hand, social interventions can have an impact on the transmission of leprosy with poverty alleviation of a more vulnerable part of the population^20^. Examples are the policy measures to mitigate poverty, such as the BFP. Corroborating with a recent study^20,25^, our data showed, in the final model, a significant reduction of the indicators of leprosy in the municipalities that had better coverage of the BFP. The BFP reached approximately 29.0% of the population of Tocantins in 2010, evidencing a scenario of poverty. Under certain conditions, the transfer of income to poor and extremely poor families can significantly increase food consumption, reduce food insecurity, improve nutritional conditions, and increase school enrollment and frequency^9,15,27^, as well as reduce barriers to access health services, especially to primary health care units.

The municipalities with populations living in poor socioeconomic conditions, high unemployment rates, probably benefited most from the BFP, as shown by a significantly lower risk for leprosy. Our and other studies suggest that interventions focused on the improvement of socioeconomic conditions may contribute to reducing the incidence of the disease^7,10,12,20^. On the other hand, in the bivariate analysis, the leprosy incidence was significantly higher in the most miserable population (higher ratio of income of the poorest 20.0%), i.e., the group of persons that are probably not part of the income transfer program with consequent difficulty to access health services. This condition leads to food shortage, proven as a socioeconomic risk factor for the clinical manifestation of the disease in different endemic areas^8,14^. Food insecurity is a result of the deprivation of basic rights, such as being alive and without disease, and being well nourished^21^.

In the State of Tocantins, 97.8% of the municipalities are small- and medium-sized (up to 50,000 inhabitants). According to data from the 2010 Census, these municipalities have a higher incidence of poverty. The average *per capita* income in the households of the most populous municipalities was more than twice as higher as that observed in municipalities with up to 50,000 inhabitants. Almost 50.0% of the population of these small municipalities lived with an average household *per capita* income of up to half minimum wage, while 24.2% lived with up to 1/4 of the minimum wage, and another 13.4% of the population lived with up to R$70.00. In large-sized municipalities, a quarter of the population lived with half minimum wage^b^. These smaller municipalities usually have a lower population density, better coverage of the BFP and FHS, and more migrants with consequent impact on the reduction of the leprosy incidence, or even a lack of trained professionals for the diagnosis.

No association was found between the leprosy incidence with urbanization rate and household density. This is due to general low household density and few populous municipalities, which are only 2.1% of the municipalities. However, there is no consensus in the literature on the association between leprosy and population density. On one hand, the endemic process of the disease can be associated with high population density, in which contact is greater^5,12^. However, on the other hand, there is no systematic evidence of this association^6,20^.

Incidence was significantly higher in municipalities with higher availability of potable water and bathroom, waste collection, and better MHDI. These variables have higher representation in areas of greater population density, more intensive movement of persons, and development, defining the predilection character of the disease^17^. Of the 139 municipalities in the State, 10 had high MHDI (> 0.7), such as Palmas, Araguaína, Gurupí, Paraíso do Tocantins, Porto Nacional, Colinas do Tocantins, Guaraí, Dianópolis, Alvorada, and Pedro Afonso. These municipalities had a population equivalent to 46.4% of the total of the State^a,b^. In fact, intra- or interregional economic growth is not translated into significant improvements in the conditions of health of a population. The situation of health cannot be explained by the total wealth of a given territory, but by the way in which it is distributed, i.e., by social inequality^26^.

The economic growth occurred in the 1970s with the installation of highway BR 153, in the 1980s with the creation of the State, and in the 1990s with the establishment of the capital and the expansion of farming activities consequently lead to the population growth of the State and improved MHDI^f^. In addition to the social problems of the resident population, these changes led to increased social problems because of the overload of the existing infrastructure, suffering from increasing demands from surrounding regions in relation to social welfare, commercial, and financial activities. In this context, the increased distribution of leprosy was related to the extension of the habitation of the the territory^17^.

Migration was another aspect associated with increased cases of leprosy in the municipalities of the State in both bivariate and multivariate analyses. In 2010, 31.5% of the population of the State consisted of immigrants, and Palmas (the capital) had a proportion of 48.1%, Araguaína of 37.1%, and Gurupi of 32.4% immigrants^b^. The migratory movements may increase the incidence of disease when susceptible migrants move to areas of high endemicity and infected migrants move to non-endemic areas, especially among the poor, who are disproportionately affected^4,18^. Migratory populations are generally more vulnerable to infectious diseases, such as leprosy. In the 1980s and 1990s, the higher number of migrants came from the endemic States of Maranhão, Goiás, Pará, and Piauí^g^. Most migrants moved from socio-economically deprived regions of high leprosy-endemicity which border the State of Tocantins^2,17,18^. Migrants usually have greater difficulty accessing health services and more commonly suffer from late diagnosis, poor housing and living conditions, and are unemployed, which define migration as an indicator of poverty and reproduction and distribution of the disease^18,19^. The fact that leprosy can be significantly higher for migrants can hinder control measures.

Although there was an increased detection rate in the bivariate analysis associated with a higher proportion of the population covered by the FHS and also higher proportion of contacts examined, this difference was not statistically significant. These actions are essential, but not sufficient for the control of the disease in a territory marked by misery and poverty^10,16,18^. On the other hand, the 139 municipalities had average coverage of the FHS above 60.0% during the 12 years of the study. There was no low coverage of the FHS to mark a possible difference^16^, as in other scenarios^7,20,23^. The increased detection of cases was associated with lower proportion of cases with grade 2 disability in the bivariate analysis. This contributes to the decrease of hidden prevalence with early diagnosis and control of the disease in municipalities with better population covered by the FHS and with trained professionals.

The observation of municipalities is fundamental for the study of the social reproduction of the disease, in which economic and cultural relations of the groups are materialized in the society. The analysis of data on leprosy in different regions is important to describe different specificities and vulnerabilities. These findings differ in some aspects from ecological studies at the national level and from other States, where the detection of cases was directly associated with municipalities with higher percentage of poverty and social inequality^5,7,12,13^.

The interpretation of the results should consider the limitations arising from the use of secondary data, which may show inconsistency in relation to the quantity and quality of the information. Potential detection errors may have overestimated the different incidences between minor and major municipalities. To minimize this limitation and correct extreme values and quiet areas, we smoothed the detection rates using the empirical Bayesian estimator^3^. Despite the mentioned limitations, the results show internal consistency and coherence with existing knowledge about leprosy and are considered representative.

The activities of control programs need to focus on municipalities of greater social vulnerability (high number of socioeconomically disadvantaged populations and migrants), with intersectoral investments for the improvement of the living conditions of the population. The good coverage of the FHS and in-service training ensure the discovery of cases, but it is not enough for an effective control. Social determinants of the disease have also to be considered.

## Footnotes

a Secretaria de Estado da Saúde do Tocantins, Coordenação de Doenças Transmissíveis e Não Transmissíveis*. Bol Epidemiológico Hanseníase* – 2013; Palmas (TO).

b Instituto Brasileiro de Geografia e Estatística. Unidades da Federação: Tocantins. Rio de Janeiro: IBGE; 2014 [cited 2014 Mar 12]. Available from: http://www.ibge.gov.br/estadosat/perfil.php?sigla=to

c Ministério da Saúde (BR), Secretaria de Vigilância em Saúde, Departamento de Vigilância Epidemiológica. Sistema de Informação de Agravos de Notificação – Sinan: normas e rotinas. 2.ed. Brasília (DF); 2007. (Série A. Normas e Manuais Técnicos).

d Ministério do Desenvolvimento Social (BR). Programa Bolsa Família (PBF). Brasília (DF); 2015. [cited 2015 Jan 7]. Available from: https://mds.gov.br/assuntos/bolsa-familia/dados

e Ministério da Saúde (BR). Portal da saúde. Histórico de cobertura da Saúde da Família. Brasília (DF); 2014 [cited 2014 Mar 4]. Available from: http://dab.saude.gov.br/portaldab/historico_cobertura_sf.php

f Ribeiro FA. A invenção do Tocantins: memória, história e representação [dissertation]. Goiânia (GO): Universidade Federal de Goiás; 2001.

g Cunha JMP, Baeninger RA, Hogan DJ. Redistribuição da população e meio ambiente: São Paulo e Centro-Oeste. Sistematização das informações censitárias sobre migração do estado do Tocantins. Campinas (SP): NEPO/Unicamp; 2000. (Sério Dados Demográficos, n. 10).
